# Antitumor effect of toosendanin on oral squamous cell carcinoma via suppression of p-STAT3

**DOI:** 10.1186/s12903-023-03602-x

**Published:** 2023-11-09

**Authors:** Ye Wu, Lingling Chen, Cheng Feng, Tao Wang, Shaohai He, Dali Zheng, Lisong Lin

**Affiliations:** 1 Fujian Key Laboratory of Oral Diseases & Stomatological Key lab of Fujian College and University, School and Hospital of Stomatology, Fujian Medical University, Fuzhou, Fujian Province, China; 2 Department of Oral and Maxillofacial Surgery, The First Affiliated Hospital of Fujian Medical University, Fuzhou, Fujian Province, China

**Keywords:** Toosendanin, Oral squamous cell carcinoma, PDX model

## Abstract

**Background:**

Toosendanin (TSN) exhibits potent antitumor activity against various tumor cell lines. However, its efficacy against oral squamous cell carcinoma (OSCC) remains unknown. Here, we investigated the effects of TSN on OSCC cells in vitro and verified them in vivo using a patient-derived xenograft (PDX) model.

**Methods:**

The effect of TSN on OSCC cells was investigated by cytotoxicity assays and flow cytometry. The expression of proteins was detected by western blotting. An OSCC PDX model was constructed to further investigate the role of TSN in regulating the function of OSCC.

**Results:**

The cell viability of CAL27 and HN6 cells decreased as the concentration of TSN increased within the experimental range. Compared with controls, TSN at lower doses inhibited cell proliferation and induced apoptosis through S-phase cell cycle arrest. TSN inhibited OSCC cell proliferation by downregulating the STAT3 pathway through the inhibition of STAT3 phosphorylation. After successful construction of the OSCC PDX model with high pathological homology to the primary tumor and treatment with an intraperitoneal injection of TSN, we showed that TSN significantly reduced the tumor size of the PDX model mice without obvious toxicity.

**Conclusions:**

Both in vitro and in vivo, TSN significantly inhibits the proliferation and promoted apoptosis of OSCC cells. Furthermore, TSN demonstrates potent inhibition of STAT3 phosphorylation, indicating its potential as a promising therapeutic agent for OSCC. Therefore, TSN holds great promise as a viable drug candidate for the treatment of OSCC.

**Supplementary Information:**

The online version contains supplementary material available at 10.1186/s12903-023-03602-x.

## Introduction

Oral squamous cell carcinoma (OSCC) is an aggressive malignancy with propensity for early metastasis and recurrence, which results in a significant reduction in overall survival. It is the predominant pathological subtype among oral neoplasms [1]. According to the National Cancer Institute (NCI), it is projected that lip and oral cavity cancer will account for approximately 54,540 new cases and 11,580 fatalities in the United States in 2023 [2]. Treatment of OSCC is challenging because of its special anatomical location and metastatic potential. The primary therapeutic modalities for OSCC encompass surgery, radiotherapy, and chemotherapy. Despite these available treatment options, the 5-year survival rate is approximately 65%, and in patients with advanced disease, the survival rate is even lower—less than 30% [3]. Despite advancements in modern medicine, there has been limited progress in reducing the mortality rate associated with OSCC. Moreover, conventional surgical treatment often fails to achieve a complete cure in patients with advanced or metastatic disease. The utilization of chemotherapy in the treatment of cancer is widely acknowledged. Chemotherapy serves as a palliative measure when tumors progress or recur but also as an adjuvant therapy alongside surgery or radiotherapy to augment the potential for achieving a complete cure [4]. Nevertheless, it is important to note that most chemotherapeutic agents used clinically exhibit nonselective cytotoxicity, affecting both tumor cells and normal cells, which often leads to undesirable adverse reactions. This limitation hampers the effectiveness of chemotherapy, emphasizing the urgent need for the development of novel therapeutic strategies and chemotherapy drugs.

In 1960, the NCI launched a program on botanical-based natural products. Since then, research has focused on utilizing highly effective and low-toxic antitumor active ingredients obtained from natural plants [5]. Over 25% of drugs are derived directly from plants, and another 25% are chemically modified natural products [6]. Botanical-based natural drugs regulate multiple pathways of tumor cell proliferation and survival, which makes them highly efficacious in the treatment of cancer. Such drugs have garnered much attention due to the availability of their active ingredients and their low biological toxicity. For example, paclitaxel, camptothecin, and vincristine are common first-line anticancer therapeutic drugs extracted from plants [7].

Toosendanin (TSN, C_30_H_38_O_11_) is a triterpenoid compound that occurs naturally in toosendan plants. TSN has traditionally been utilized for its effectiveness in eliminating ascariasis within the digestive tract [8]. The pharmacological effects of TSN are extremely broad and include selective blockade of acetylcholine release from nerve endings and significant anti-carnitine activity [9]. Furthermore, TSN exhibits notable inhibitory properties against tumor cell proliferation in diverse cancer types, including hepatocellular carcinoma, prostate cancer, colorectal cancer, and breast cancer [10]. Compared with similar chemotherapy drugs, TSN has a lower 50% inhibitory concentration (IC_50_), which indicates a more potent inhibitory effect on cancer cells [11, 12]. TSN demonstrates a multitarget regulatory effect on diverse tumor cell lines, effectively suppressing tumor cell proliferation by modulating downstream signaling pathways [13]. Thus, it holds promising prospects as an anticancer agent. A previous study [14] has documented the molecular-level inhibitory effects of TSN on the proliferation of breast tumor cells, which possibly involve the induction of necrosis, apoptosis, and autophagy of tumor cells. In addition to its standalone inhibitory effect on tumor cell proliferation, TSN has a sensitizing effect on conventional chemotherapy drugs and can even reverse drug resistance. For example, TSN may mediate the sensitization of NSCLC cells to cisplatin by downregulating the expression of Anxa4 [15]. Another study [16] found that TSN reversed the resistance of breast cancer cells to adriamycin via inhibiting PI3K, suggesting that the combination of adriamycin and TSN shows great potential for effectively treating human breast cancer. TSN shows promising antitumor potential, but it is still unknown whether it has inhibitory effects on OSCC cells.

In recent years, patient-derived xenograft (PDX) models have gained significant research attention [17, 18]. Such models have demonstrated promising outcomes in tumor drug screening and clinical translational research, establishing themselves as a valuable tool for preclinical tumor investigations [19]. Unlike conventional cell line–derived xenograft (CDX) models, PDX models faithfully capture the heterogeneity of the primary tumor tissue and recreate a microenvironment that is difficult to simulate adequately [20]. Consequently, such models offer a reliable representation of tumor behavior and response, particularly in terms of assessing tumor drug efficacy and response in a manner that closely resembles that observed in patients [21, 22]. PDX models have emerged as prominent tumor models employed in cancer research and are particularly favored by the NCI. PDX models involve the transplantation of tumor tissues from cancer patients into immunocompromised mice, offering a preferred approach for studying cancer and anticancer therapeutic interventions [23].

In summary, TSN has strong antitumor potential, which makes it a promising candidate for a chemotherapeutic agent. However, it also has the disadvantages of poor water solubility and low bioavailability, and its effect on OSCC has not been determined. Therefore, in this study, we used nude mice to construct a PDX model to investigate the effects of TSN on the growth and progression of OSCC and determined the therapeutic effects of TSN in vivo and in vitro.

## Materials and methods

### Cell culture and reagents

Human oral epithelial cells (HOECs) and OSCC (CAL27 and HN6) cell lines were obtained from Fujian Key Laboratory of Oral Diseases and cultured in high-glucose DMEM (Gibco, Grand Island, NY, USA) supplemented with 10% fetal bovine serum (FBS, SORFA, Beijing, China). The cells were cultured at 37 °C in a 5% CO_2_ humidified incubator.

### Materials

TSN and 5-fluorouracil were purchased from MedChemExpress (MCE, Shanghai, China). Dimethyl sulfoxide (DMSO) was purchased from Sigma Aldrich (St. Louis, MO, USA). We dissolved 1 mg TSN in 1.7403 mL DMSO to prepare a TSN solution (1 mmol/mL), and we stored the solution at − 80 °C for subsequent utilization. Antibodies against p-STAT3 (Tyr705), STAT3, and GAPDH were purchased from Cell Signaling Technology Inc. (Danvers, MA, USA). Antibodies against Ki-67, CK5/6, P53, and P16 were purchased from HuaBio Inc. (Shanghai, China). BALB/C-nude were provided by SLAC Laboratory Animal Co., Ltd (Shanghai, China).

### Cell survival assay

The concentration of TSN producing 50% growth inhibition (IC_50_) was measured by the CCK-8 method. Cells were seeded in 96-well plates (5 × 10^3^ cells per well) and cultured at 37 °C with 5% CO_2_ for 12 h. TSN was dissolved in DMSO (1 mmol/mL), and then, the TSN solution was diluted by adding it to DMEM to formulate TSN solutions of different concentrations (final concentrations of 0, 0.001, 0.01, 0.1, 1, and 10 μM). Next, samples of the different TSN solutions (100 μL) were added to the plates. After 48 h, each well was incubated with 100 μL of a specialized culture medium containing CCK-8 (10%) at 37 °C in 5% CO_2_ for 1 h. The absorbance was detected at 450 nm with a microplate reader (MolecularDevices, San Jose, CA, USA). The IC_50_ of TSN was calculated from the dose–response curve.

The effect of TSN on OSCC proliferation was determined by the CCK-8 assay, with an equivalent volume of DMSO included as a control. The concentrations of TSN in the experimental group were 5 nM and 10 nM, in accordance with the IC_50_ values obtained in the previous experiment. OSCC cells (CAL27 and HN6 cells) were cultured in 96-well plates (5 × 10^3^ cells per well) at 37 °C with 5% CO_2_. After 0, 24, 72, and 120 h, 100 μL of a specialized culture medium containing CCK-8 (10%) was added, and the mixture was incubated at 37 °C in 5% CO_2_ for 1 h. The absorbance of each well at 450 nm was measured. Three replications of all experiments were conducted. Each treatment's effect was evaluated as a percentage of cell survival, with untreated control cells considered to be 100% alive [24].

### Annexin V-FITC/PI staining

The Annexin V-FITC/PI fluorescence detection kit (Uelandy, Shanghai, China) was employed for the detection of apoptosis. OSCC cells (CAL27 and HN6 cells, 3 × 10^5^ cells per well) were seeded in six-well plates, TSN solutions (5 nM and 10 nM) were added to the wells, and the mixtures were incubated at 37 °C in 5% CO_2_ for 48 h. The collected cells (1 × 10^5^ cells per tube) were subjected to centrifugation at 200 g for 5 min and resuspended in 100 μL of buffer containing 5 μL Annexin V-FITC and 5 μL PI. Subsequently, the cells were stained in darkness at − 4 °C for 15 min and analyzed by flow cytometry (Becton, Dickinson and Company, LSRFortessaX-20, NJ, USA).

### Cell cycle assay

The effect of TSN on cell cycle was assessed using a cell cycle and apoptosis kit (Uelandy, Shanghai, China). HN6 cells were placed in six-well plates (3 × 10^5^ cells per well) and treated with TSN (5 nM or 10 nM) for 48 h. Flow cytometry was utilized to analyze the distribution of cells among the stages of cell cycle, and the distribution of cells in G0/G1, S, or G2/M phases was assessed by measuring the respective regions.

### Western blot

OSCC cells (CAL27 and HN6 cells, 3 × 10^5^ cells per well) were treated with TSN (0, 5, 10, and 20 nM) for 48 h and then lysed with 200 μL RIPA buffer (Sigma-Aldrich, St. Louis, MO, USA) containing 1% PMSF (Beyotime, Shanghai, China). Next, the total proteins were collected and their concentration was determined by the Bicinchoninic Acid (BCA) method (Beyotime, Shanghai, China). The proteins of each sample were separated by 12% SDS-PAGE (100 V, 90 min) and transferred to PVDF (Cytiva, MA, USA) membranes (100 V, 100 min). Prior to incubation with primary antibodies, the blots were cut into two or three sections. After blocking in 3% bovine serum albumin for 1 h at roomtemperature, the membranes were incubated with primary antibodies against STAT3 (1:1000), p-STAT3 (1:1000), and GAPDH (1:1000) at 4 °C for 10 h and with goat anti-rabbit horseradish peroxidase (HRP) secondary antibodies for 1 h at room temperature. Finally, the membranes were soaked in ECL luminous solution and detected using ChemiDoc imaging system (Bio-Rad, CA, USA). The gray values of proteins were measured using ImageJ.

### In vivo* study of the PDX model*

The tumor specimens used in this study were provided by the Affiliated Hospital of Fujian Medical University (Approval Number: FJMU-IACUC 2021–0461), and written informed consent was obtained from each participant. A part of the tumor resected from the patient was immediately stored in DMEM. Then, 2–3-mm^3^ tumor blocks were implanted into the groins of 4-week-old male Balb/c nude mice (F1). When the F1 tumor grew to about 100 mm^3^ in size, it was transplanted into the second-generation Balb/c nude mice (F2). Similarly, when the F2 tumor grew to 100 mm^3^ in size, it was transplanted into the third-generation Balb/c nude mice (F3). The F3 mice were randomly divided into two different treatment groups and a blank control group, with seven mice in each group. The drugs 5-FU and TSN were initially dissolved in DMSO to achieve a concentration of 1 mM and subsequently diluted to a concentration of 1 mg/mL using saline. Intraperitoneal injection was administered at a dosage of 5 mg/kg, taking into consideration the weight of the mice. The mice were administered an intraperitoneal injection of 5-FU (5 mg/kg) and TSN (5 mg/kg) for 4 weeks. We calculated the volume of the tumor using the following Eq. 1 [25]:

1 $$\mathrm{Tumor}\;\mathrm{volume}\:=\:\mathrm{length}\:\times\:\mathrm{width}^2\:\times\:0.5$$

### Histological examination

The tumor tissue was fixed in a 4% formaldehyde solution for 24 h. Subsequently, the tissue was embedded in paraffin and sliced into sections with a thickness of 4 μm. The sections were then dehydrated, and Hematoxylin and Eosin (H&E) staining, immunohistochemical (IHC) staining and TUNEL staining were performed on the sections in line with the instructions provided in the kit (Uelandy, Shanghai, China). Furthermore, after blocking with 1% normal goat serum, the tumor slices were incubated with antibodies against Ki-67 (1:500), CK56 (1:200), P53 (1:500), P16 (1:200), and p-Stat3 (1:100) at 4 °C for 10 h. Finally, the sections were treated with HRP-conjugated antibody at room temperature for 1 h. The examination and imaging capturing were conducted using a fluorescence inverted microscope (Zeiss, Oberkochen, Bavaria, Germany).

### Statistical analysis

Statistical analysis was performed using Prism9 software (GraphPad, San Diego, CA, USA). The IC_50_ value was calculated using nonlinear regression for dose–response inhibition. For comparisons between two groups, we used Student's t tests, and for comparisons among three groups, we used one-way ANOVA. The data are expressed as mean ± standard deviation (SD). *p* value lower than 0.05 was considered statistically significant.

## Results

### TSN inhibits the proliferation of OSCC cells

**Fig. 1:**
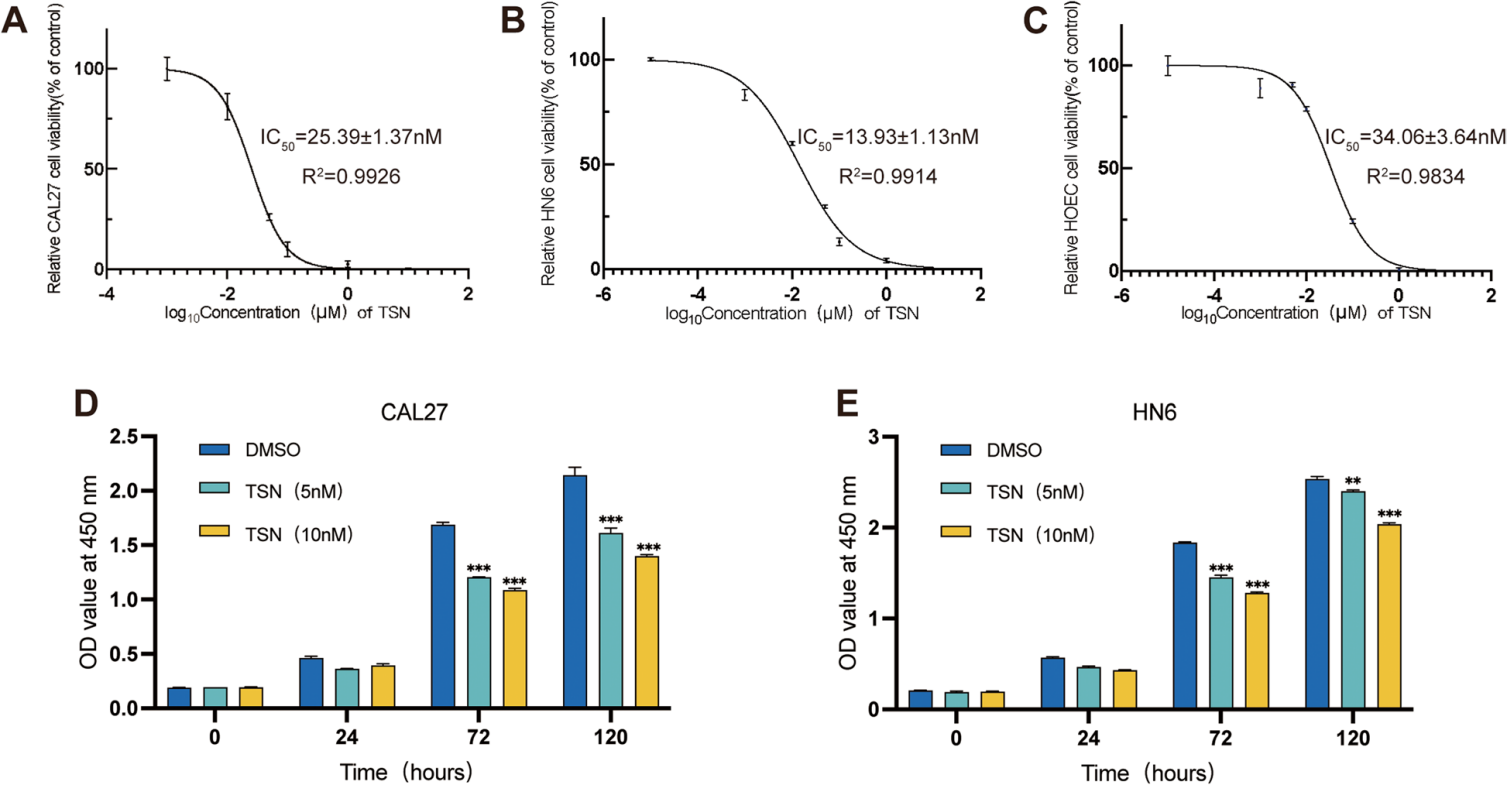
Effect of Toosendannin (TSN) on cell viability and proliferation of OSCC cells. Fitted curves for CAL27 (**A**), HN6 (**B**), and HOEC (**C**) cell viability–concentration after 48 h of TSN treatment. The cell proliferation ability of CAL27 (**D**) and HN6 (**E**) cells treated with TSN. **p < 0.01, ***p < 0.01 compared with the corresponding 72-h and 120-h DMSO groups. Data are expressed as the mean ± SD

The viability of CAL27, HN6, and HOEC cells after 48 h of TSN treatment was assessed by the CCK-8 method. The IC_50_ values were calculated by fitting the dose–response curve. The results showed that the IC_50_ values of TSN on CAL27, HN6, and HOEC cells were 25.39 ± 1.37 nM, 13.93 ± 1.13 nM, and 34.06 ± 3.64 nM, respectively (Fig. 1A, B, C). Notably, TSN had a higher IC_50_ for HOEC, suggesting that TSN is less toxic to normal oral cells. Considering that the IC_50_ value of TSN on HN6 cells was13.93 ± 1.13 nM, the concentrations of 5 nM and 10 nM were selected for the subsequent experiments to avoid excessive cell death resulting from excessive drug toxicity.

**Fig. 2:**
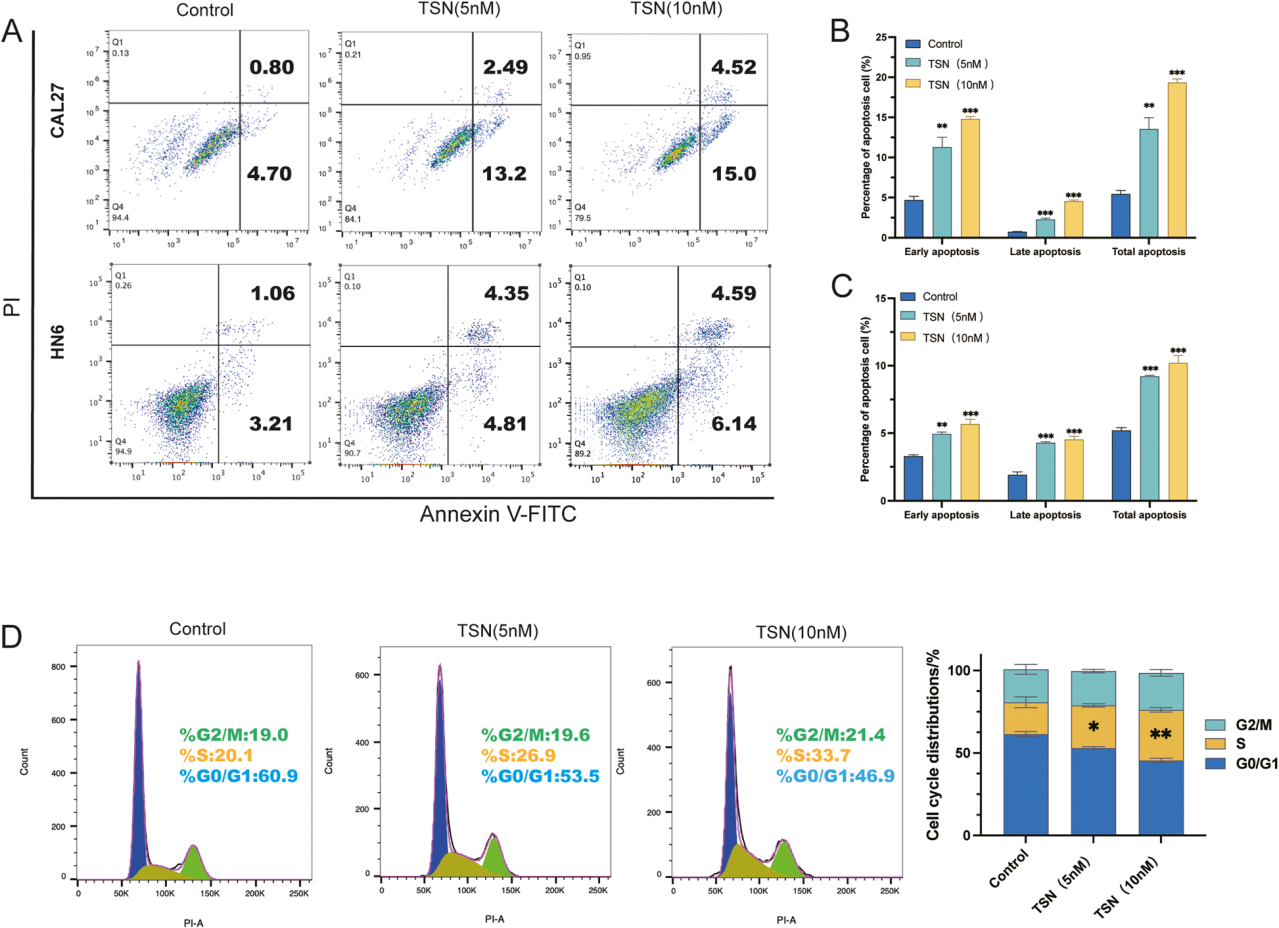
TSN induces apoptosis and triggers S-phase cell cycle arrest in OSCC cells. **A** Flow cytometry analysis of apoptosis of CAL27 and HN6 cells. **B** Apoptosis percentage of CAL27 cells after treatment with TSN. **C** Apoptosis percentage of HN6 cells after treatment with TSN. **D** TSN induces S-phase cell cycle arrest in OSCC cells. (n = 3) *p < 0.05, **p < 0.01, ***p < 0.001 compared with the control group

To assess the inhibitory effects of TSN on the proliferation of OSCC cells, the cell viability of these cell lines was assessed at different time points (0, 24, 72, and 120 h) upon treatment with different concentrations of TSN (5 and 10 nM). Figure 2D and E shows the effect of TSN on the proliferation of CAL27 and HN6 cells. Notably, the growth patterns of cells in the TSN and DMSO groups began to diverge after 72 and 120 h. Moreover, within a specific concentration range, the proliferation activity of OSCC cells gradually decreased with increasing TSN concentration and prolonged exposure time.

### TSN induces apoptosis and triggers S-Phase cell cycle arrest in OSCC cells

Previous studies have shown that TSN can induce apoptosis of various tumor cell lines. To investigate the potential of TSN to induce apoptosis in CAL27 and HN6 cells, we treated the cells with TSN for 48 h. Flow cytometry analysis (Fig. 2A) demonstrated an increase in the apoptosis rate of CAL27 and HN6 cells after TSN treatment. These findings suggest that TSN has the ability to induce both early and late apoptosis in CAL27 and HN6 cells. The total apoptosis rates of CAL27 cells after treatment with 5 nM TSN and 10 nM TSN were (13.56 ± 2.42)% and (19.36 ± 0.75)%, respectively, which were significantly higher than the apoptosis rate of the DMSO group (5.45 ± 0.78)% (Fig. 2B). The total apoptosis rates of HN6 cells in the TSN groups with concentrations of 5 nM and 10 nM were (9.22 ± 0.82)% and (10.21 ± 0.97)%, respectively, which were also significantly different from the DMSO group(5.20 ± 0.35)% (Fig. 2C).

Flow cytometry analysis demonstrated that TSN could induce apoptosis in OSCC. Based on this finding, we conducted a cell cycle assay on the OSCC cells treated with TSN. The results revealed a decreased proportion of cells in the G0/G1 phase and an increased proportion of cells in the S phase after 48 h of 5 nM (*p* < 0.05) and 10 nM (*p* < 0.01) TSN treatment compared with the control group (Fig. 2D). Taken together, TSN may inhibit the proliferation of OSCC cells and promote their apoptosis by triggering S-phase cell cycle arrest.

### TSN inhibits STAT3 phosphorylation level

**Fig. 3:**
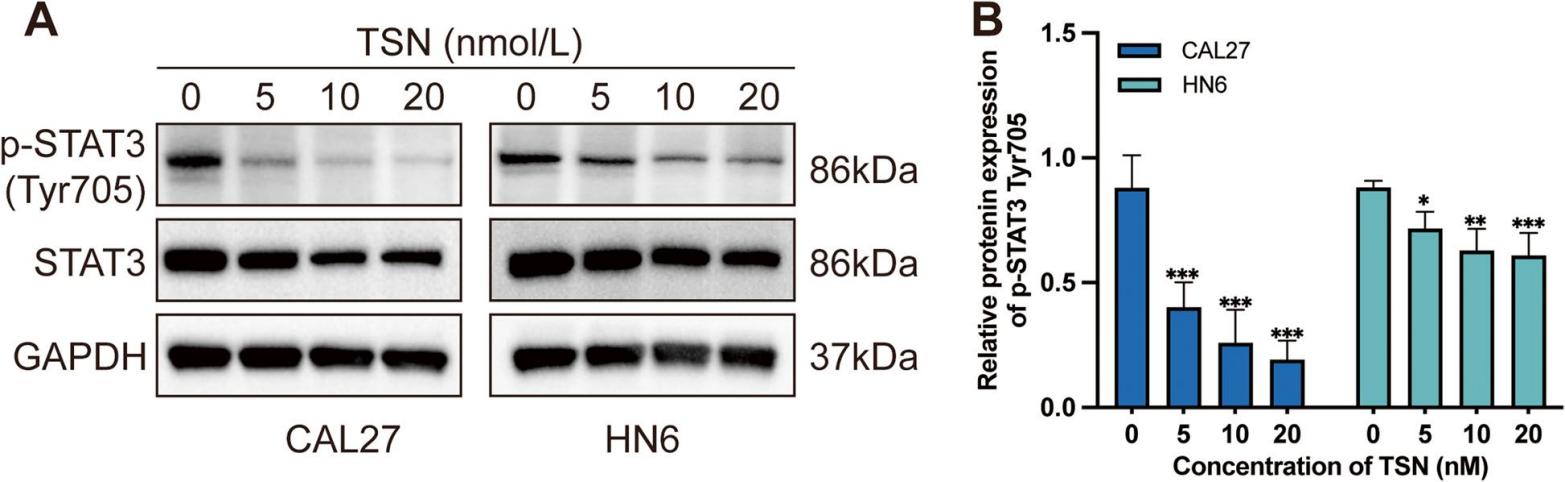
TSN induces OSCC apoptosis via p-STAT3 inhibition. **A** Western blot analysis of total STAT3 and p-STAT3 in OSCC cells (CAL27 and HN6) after treatment with TSN. **B** Relative expression of p-STAT3 (Tyr705) protein in OSCC cells (*n* = 4). *p < 0.05, **p < 0.01, ***p < 0.001 compared with the 0 nM (control) group. The blots were cut prior to hybridization with antibodies. Full-length blots are presented in Supplementary Fig. 1

STAT3 is a transcription factor that plays a crucial role in the growth and proliferation of cells, including tumor cells. Activation of STAT3 can promote cell survival, proliferation, and angiogenesis, thereby contributing to the development and progression of cancer. Inhibiting the expression of p-STAT3, which is the phosphorylated form of STAT3, can induce apoptosis in tumor cells. We investigated the inhibitory effect of TSN on the STAT3 pathway and its role in promoting apoptosis in OSCC cells. Phosphorylation levels of STAT3 were assessed by western blot analysis. The results (Fig. 3A) showed a significant reduction in the level of STAT3 phosphorylation (Fig. 3B) following TSN treatment in both CAL27 and HN6 cells. Compared with the control group, CAL27 cells particularly showed a lower level of p-STAT3 expression (*p* < 0.001). As shown in Fig. 3B, the low concentration of TSN (5 nM) caused a decrease in the phosphorylation level of STAT3 in OSCC cells, preliminarily suggesting that TSN may be a promising inhibitor of STAT3 phosphorylation.

### In Vivo* antitumor efficiency of TSN in  the PDX model*

**Fig. 4:**
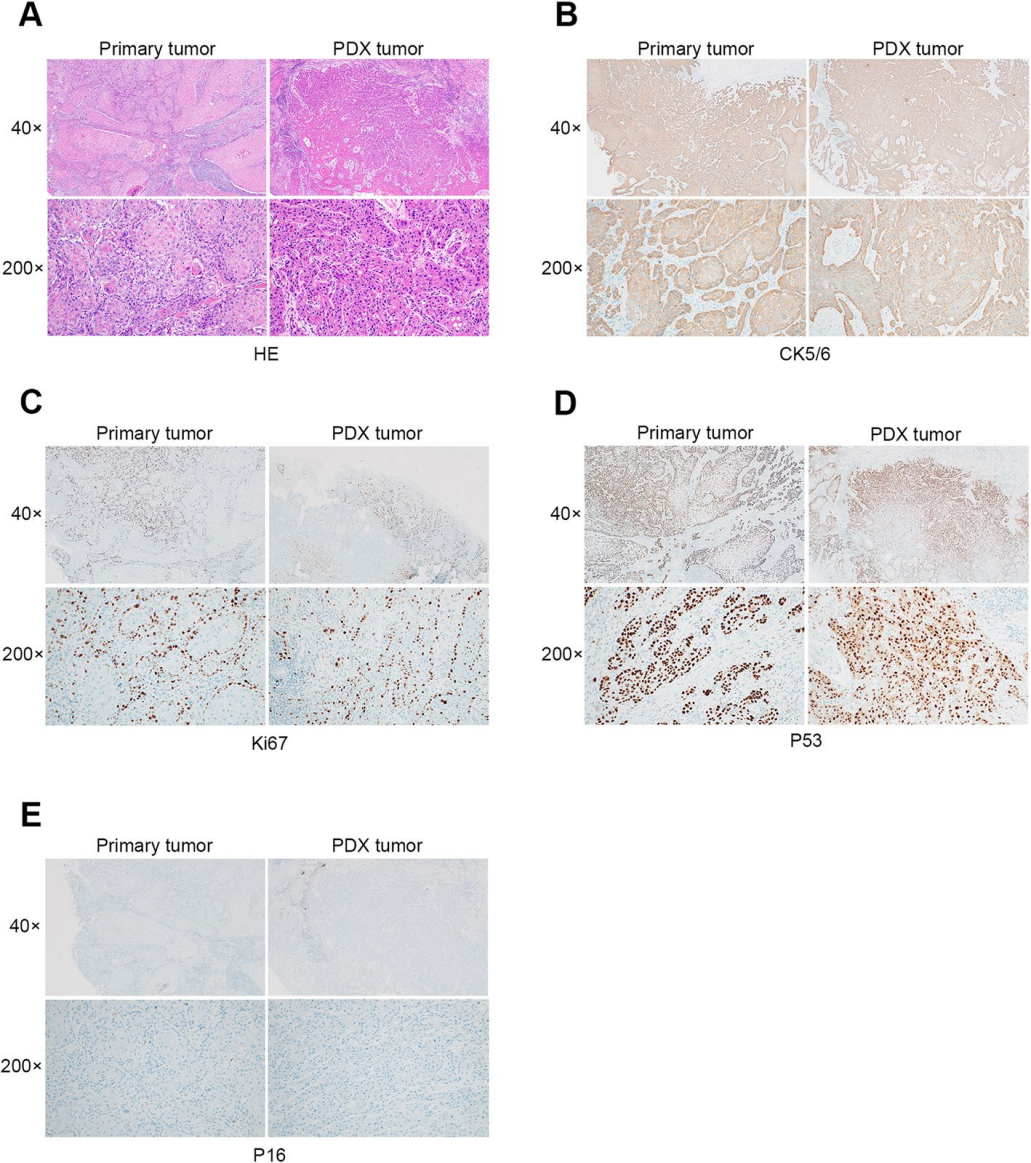
Histopathological results of the primary tumor and the PDX tumor. (**A**) H&E staning (**B**) CK5/6; (**C**) Ki67; (**D**) P53; (**E**) P16. The positive control of P16 IHC staning was presented in Supplementary Fig. 2

In our previous in vitro study, we demonstrated the efficacy of TSN in inhibiting the growth of OSCC cells. To further validate these findings in an in vivo model, we established a PDX model. The primary tumor and the PDX tumor were subjected to H&E staining, and immunohistochemical analysis was performed (Fig. 4). The results confirmed the successful establishment of the PDX model, which exhibited a high degree of pathological similarity to the patient's tumor.

**Fig. 5:**
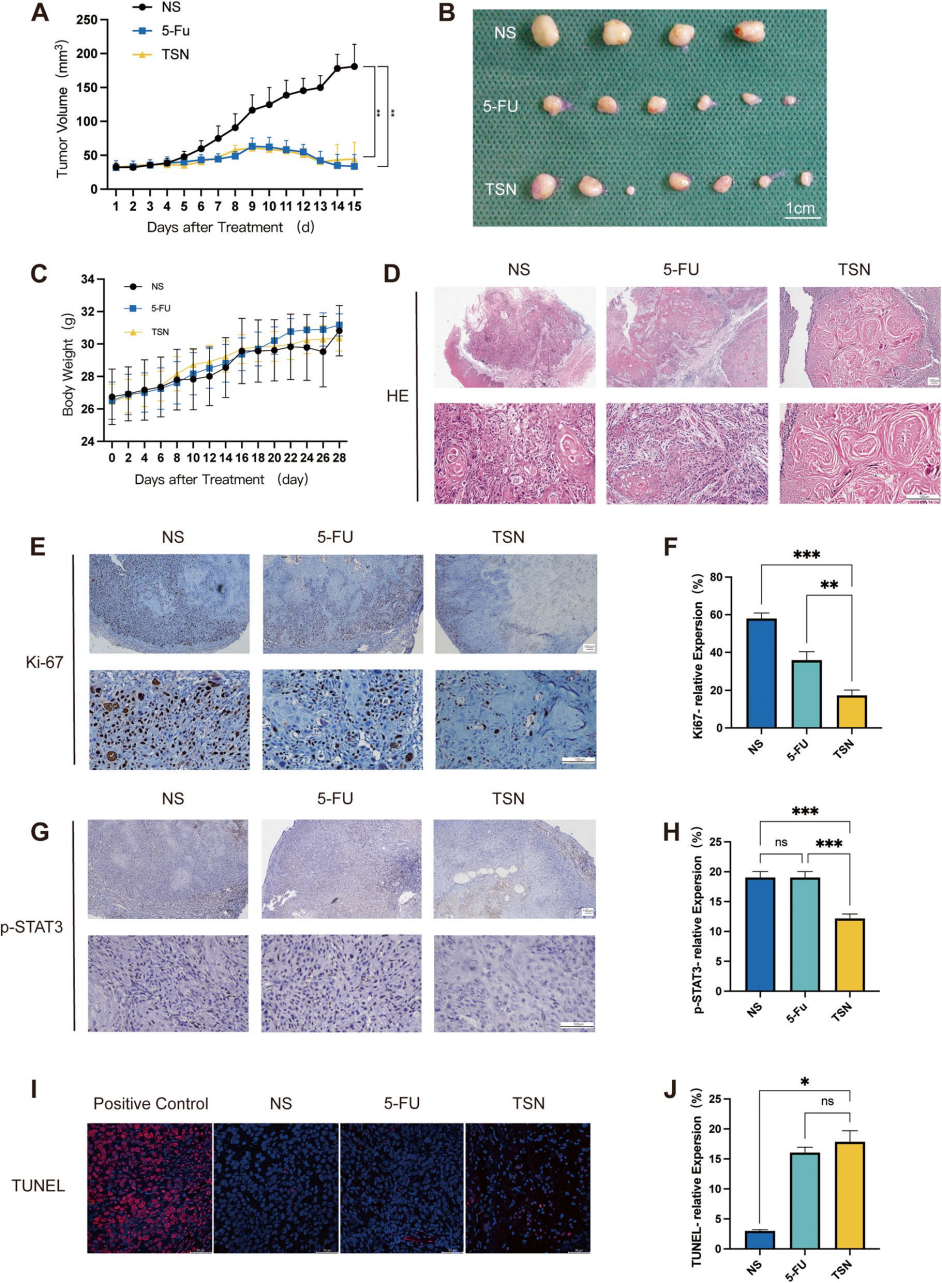
TSN inhibits tumor progression of OSCC in the PDX model. **A** Tumor volume of each group. **p < 0.01 compared with the NS (normal saline) group. **B** Tumor images of each group of mice at the end of the experiment. **C** Body weight of the mice in each group. **D** H&E staining of the tumor tissues. **E**, **F** Immunohistochemistry representative image and statistical analysis of Ki67 in the tumor tissues. **p < 0.01,***p < 0.01. **G**, **H** Immunohistochemistry representative image and statistical analysis of p-STAT3 in the tumor tissues. ^*ns*^ (not significant)* p* > 0.05*,* ***p < 0.001. **I**, **J** TUNEL staining and statistical analysis of the tumor tissues. ^*ns*^* p* > 0.05, *p < 0.05. Data are expressed as the mean ± SD

When the tumor volume had increased to approximately 100 mm^3^, the mice were randomly divided into three groups, with seven mice in each group. Following a 28-day intraperitoneal injection treatment, three mice in the NS (normal saline) group and one mouse in the 5-FU group died, and we speculated that the cause of death might be due to excessive tumor size or infection. Throughout the experiment, the change in body weight among the three groups was insignificant, indicating that TSN exhibited no apparent toxicity to the mice (Fig. 5C). Subsequently, the tumors derived from these mice were excised for further analysis (Fig. 5B). As shown in Fig. 5A, both the TSN group and the 5-FU group demonstrated significantly lower tumor volumes compared with the NS group, indicating a statistically significant reduction in tumor size (*p* < 0.01). However, there was no significant difference between the 5-FU and TSN groups (*p* > 0.05).

H&E staining (Fig. 5D) showed that the tumor cells in the NS group were round and closely arranged, with large deep-stained nuclei, showing obvious hypo-differentiation. IHC staining revealed that TSN reduced the expression of p-STAT3 (Fig. 5H), which was consistent with the results of western blot. The apoptotic and proliferative status of the tumor cells was evaluated through Ki67 and TUNEL staining of the tumor tissues (Fig. 5E, I). As shown in Fig. 5F, there was a noteworthy decrease in Ki-67 expression in the TSN group compared with that in the NS group (*p* < 0.001). Similarly, there was an elevated TUNEL signal (Fig. 5I, J), indicative of increased apoptosis (*p* < 0.05). Taken together, these results suggest that TSN may be effective in inhibiting STAT3 phosphorylation and thus in inhibiting OSCC growth.

## Discussion

TSN has widely been investigated for its ability to promote apoptosis in various tumor cell lines and enhance the effectiveness of chemotherapy drugs against drug-resistant tumors. These findings suggest that TSN holds promise as a potential therapeutic agent for combating different types of cancer [26–28]. However, the precise effect of TSN on OSCC cells and the underlying mechanism remain unclear. Therefore, in this study, CAL27 cells, HN6 cells, and an OSCC-related PDX model were employed to conduct in vivo and in vitro experiments, aiming to validate the effect of TSN on OSCC.

TSN demonstrated remarkable inhibitory effects on the growth of OSCC cells even at very low concentrations. It exhibited cytotoxicity and induced cell apoptosis in a time- and dose-dependent manner. Flow cytometry analysis revealed that TSN effectively induced apoptosis in OSCC cells, consistent with previous studies on other tumor types. These results further support the potential of TSN as an anticancer agent with broad applicability across different cancers [29].

Our western blot results showed that the level of p-STAT3 was markedly reduced in the TSN group. STAT3 is a special transcription factor that is widely present in the cytoplasm. It regulates genes involved in cell growth, metastasis, and angiogenesis; is associated with tumor cell proliferation; and is an emerging target for cancer therapy [30]. They can be phosphorylated to form homodimers or heterodimers, thus forming activating transcription factors p-STAT3. p-STAT3 possess the capacity to traverse the nucleus and selectively bind to specific sites, thereby exerting regulatory control over the transcriptional activity of downstream genes. Consequently, they play a pivotal role in modulating diverse cellular physiological processes, including proliferation, differentiation, apoptosis, angiogenesis, and immune regulation [31]. The STAT signaling pathway constitutes a rapid membrane-to-nucleus signaling module and induces the expression of key mediators of various cancers and inflammation [32]. Under typical conditions, STAT3 activation is rapid and transient because of the presence of several negative regulators within the cell. However, previous studies have found constitutive activation of STAT3 in various tumor cell lines and human tumor tissues, and STAT3 activation is considered a marker of poor tumor prognosis [33, 34]. A previous study [35] showed that TSN could inhibit osteosarcoma growth by targeting STAT3. The possible mechanism is that TSN can directly bind to the SH2 structural domain of STAT3, inhibit the activation of STAT3 phosphorylation, and reduce p-STAT3 expression levels to inhibit tumor cell proliferation. In line with this finding, several recent studies have shown that STAT3 is overexpressed and constitutively activated in OSCC. It plays an important role in OSCC invasiveness [36, 37]. STAT3 can promote OSCC cell proliferation and inhibit apoptosis by increasing the expression of target genes such as Mcl-1, c-Myc, Glut5, cyclin D1, Bcl-2, and Bcl-xL [38–41]. Consequently, STAT3 has emerged as a prospective molecular target and biomarker for OSCC, with STAT3 inhibitors exhibiting effectiveness in restraining tumor growth and metastasis in OSCC. This further demonstrates the great potential of TSN in inhibiting the proliferation of OSCC cells, and it is of great clinical significance to develop TSN and its derivatives as potential STAT3 inhibitors.

In the present study, we aimed to assess the efficacy of TSN in inhibiting the PDX model of OSCC in vivo. Traditional approaches for cancer drug screening involve the cultivation of human tumor cell lines, followed by their transplantation into immunodeficient mice. However, these models are limited by their artificial nature, as the tumor cell lines are often cultured under optimal conditions, which may not accurately reflect the genetic and epigenetic heterogeneity present in primary tumors [42, 43]. In contrast, PDX models preserve the differentiation, structural, and molecular biological features of the primary tumor, which faithfully recapitulates the in vivo tumor microenvironment. Thus, PDX models could enhance the precision in predicting the therapeutic efficacy of drugs and their antitumor effects [44, 45]. Hence, we chose the PDX model to assess the effect of TSN on OSCC in vivo. Our PDX model demonstrated a high degree of pathological similarity with the original tumor, as demonstrated through the immunohistochemical analysis of the PDX tumor mass and the primary tumor. We showed that the effects of TSN on the PDX model mice closely resembled those of 5-FU. The results of p-STAT3 immunohistochemical staining of the tumor tissue sections showed that the expression of p-STAT3 was significantly reduced in the tumor tissues of the TSN group, compared with that in the NS and 5-FU groups. Subsequently, we showed that TSN might inhibit tumor development by regulating the phosphorylation level of STAT3. Consequently, it can be inferred that TSN exhibits a favorable effect on the chemotherapeutic efficacy in OSCC.

**Fig. 6:**
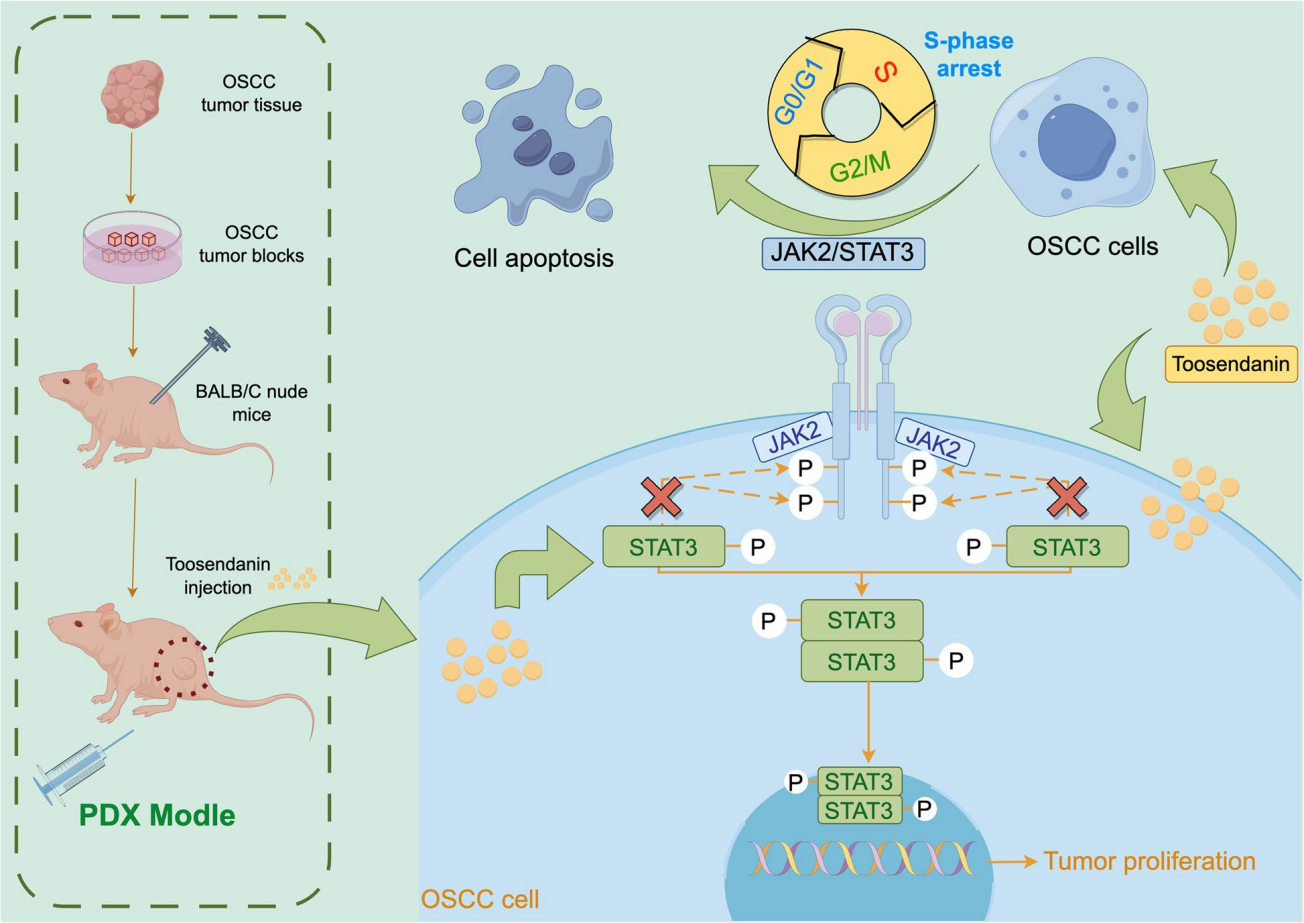
TSN induced proliferation and promoted apoptosis by arresting the S-phase cell cycle and inhibiting STAT3 phosphorylation levels of OSCC in vivo and in vitro studies. This figure was draw by Figdraw

In conclusion, as shown in Fig. 6, our results from both in the PDX model and in vitro studies suggest that TSN may have the potential to induce proliferation and promote apoptosis by arresting the S-phase cell cycle and inhibiting STAT3 phosphorylation levels in OSCC. Therefore, TSN may serve as a potential STAT3 phosphorylation inhibitor, providing a new direction for the study of OSCC chemotherapeutic agents. However, it is important to acknowledge that our study is a preliminary investigation into the role of TSN in OSCC, and thus, it has certain limitations. The inherent properties of TSN, including low water solubility, low stability, and low bioavailability, also impose limitations on its application [46]. Consequently, despite the practical implications of our findings, additional experiments are imperative to assess the efficacy of TSN in the treatment of OSCC.

## Conclusion

Both in vitro and in vivo, TSN exhibits a noteworthy inhibitory effect on OSCC growth by suppressing p-STAT3, which indicates its potential as a STAT3 phosphorylation inhibitor. In summary, TSN could be an effective anticancer candidate for treating OSCC.

### Supplementary Information

**Additional file 1: Supplementary Figure 1. **The original full-length blots of western blot experiments.** Supplementary Figure 2. **Positive control of P16 IHC staining.

## Data Availability

The datasets used and analysed during the current study are available from the corresponding author on reasonable request.

## Abbreviations

TSN: Toosendanin
OSCC: Oral squamous cell carcinoma
SD: Standard deviation
PDX: Patient-derived tumor xenograft
CCK-8: Cell Counting Kit-8
5-FU: 5-Fluorouracil
