# Supplementary material for: Antitumor effect of toosendanin on oral squamous cell carcinoma via suppression of p-STAT3
Source: BMC Oral Health. 2023 Nov 9;23:846. doi: 10.1186/s12903-023-03602-x (PMC10634166; doi:10.1186/s12903-023-03602-x)
Supplement: Supplementary file 1 — Additional file 1: Supplementary Figure 1. The original full-length blots of western blot experiments. Supplementary Figure 2. Positive control of P16 IHC staining. [file 12903_2023_3602_MOESM1_ESM.docx]

**Manuscript Title：**

Antitumor Effect of Toosendanin on Oral Squamous Cell Carcinoma via Suppression of p-STAT3

**Author:**

Ye Wu^1^, Lingling Chen^1^, Cheng Feng^1^, Tao Wang^1^, Shaohai He^1^ , Dali Zheng^1,*^, Lisong Lin^2,*^

^1^Fujian Key Laboratory of Oral Diseases & Stomatological Key lab of Fujian College and University, School and Hospital of Stomatology, Fujian Medical University, Fuzhou, Fujian Province, China

^2^ Department of Oral and Maxillofacial Surgery, The First Affiliated Hospital of Fujian Medical University, Fuzhou, Fujian Province, China

^*^ Correspondence should be addressed to Dali Zheng (dalizheng@fjmu.edu.cn) and Lisong Lin (dr_lls@fjmu.edu.cn)


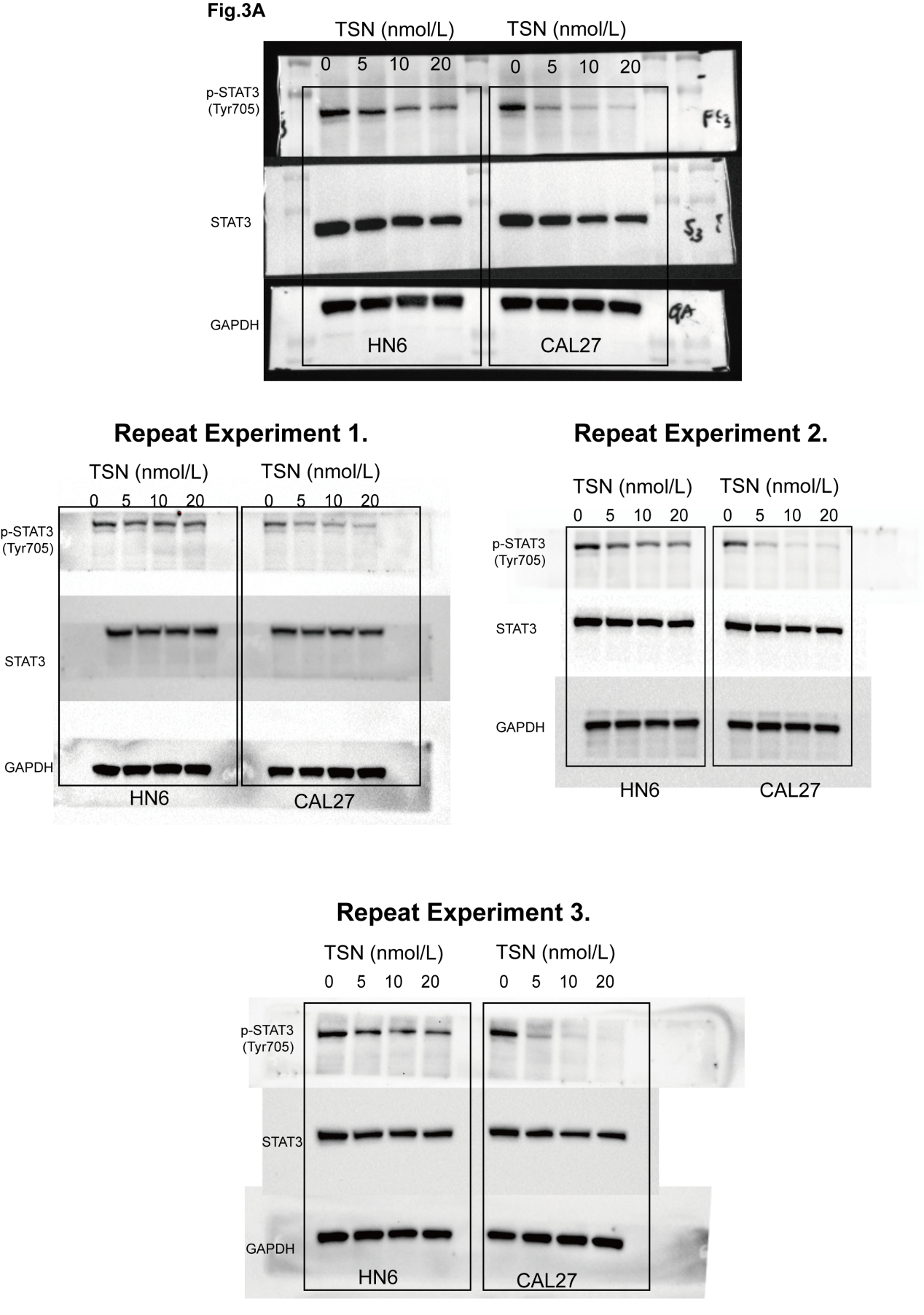


**Supplementary Figure 1.** The original full-length blots of western blot experiments.


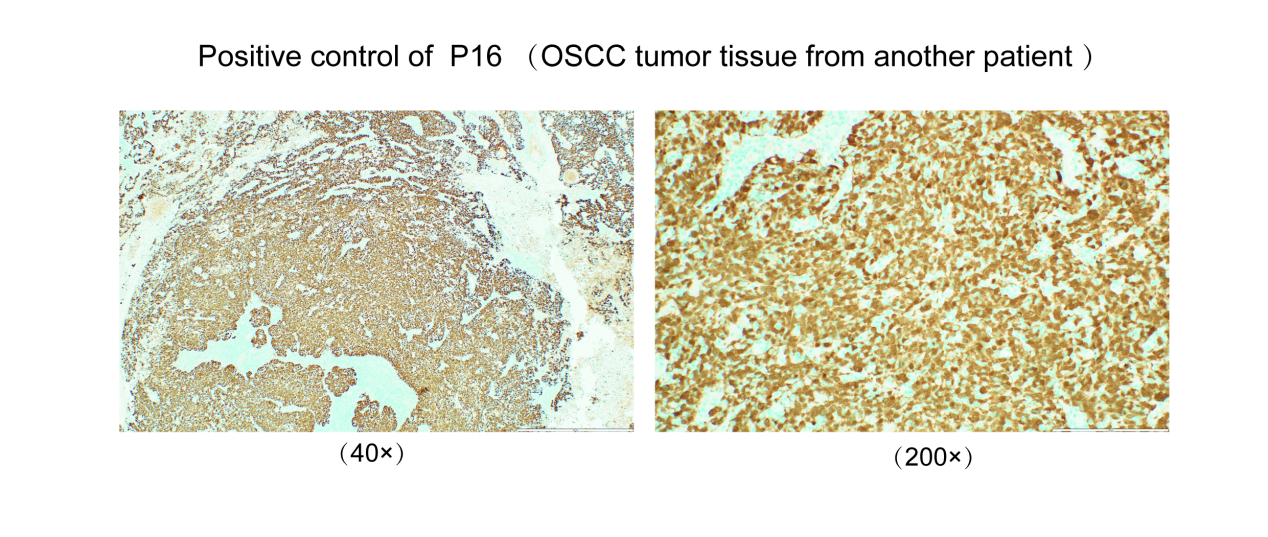


**Supplementary Figure 2.** Positive control of P16 IHC staining.
